# YouthCHAT as a Primary Care E-Screening Tool for Mental Health Issues Among Te Tai Tokerau Youth: Protocol for a Co-Design Study

**DOI:** 10.2196/12108

**Published:** 2019-01-09

**Authors:** Rhiannon Mary Martel, Margot Louise Darragh, Aniva Joanne Lawrence, Matthew John Shepherd, Tracey Wihongi, Felicity Anne Goodyear-Smith

**Affiliations:** 1 Department of General Practice and Primary Health Care Faculty of Medical and Health Science The University of Auckland Auckland New Zealand; 2 Manaia Health Primary Health Organisation Whangarei New Zealand; 3 Te Tai Tokerau Primary Health Organisation Kerikeri New Zealand; 4 Faculty of Education and Social Work Department of Counselling, Human Services and Social Work The University of Auckland Auckland New Zealand; 5 Ngati Hine Health Trust Whangarei New Zealand

**Keywords:** adolescents, brief intervention, co-design, mental health, primary health care, protocol, risk behavior, YouthCHAT

## Abstract

**Background:**

In New Zealand (NZ), 1 in 4 adolescents is affected by mental health issues (eg, depression and anxiety) and engages in risk behaviors (eg, harmful drinking and substance abuse), with rates among Māori youth being significantly higher. The majority of NZ secondary school students visit their local primary health care providers (PHPs) at least annually, yet most do not seek help for mental health and risk behavior (MHB) concerns. While youth think it acceptable to discuss sensitive issues during a consultation with their PHPs, unless problems are severe, such conversations are not initiated by PHPs. Early intervention for MHB concerns can prevent long-term health and well-being issues. However, this relies on the early identification of developing problems and youth being offered and accepting help. YouthCHAT is an electronic, multi-item screening tool developed in 2016 to assess MHB concerns among youth. YouthCHAT is completed before a consultation with the PHP, who can access a summary report straight away. A help question allows young people to identify issues that need addressing. A resource pack uses stepped care pathways to guide providers to use appropriate brief interventions.

**Objective:**

This study aimed to explore the utility, feasibility, and acceptability of YouthCHAT when tailored for use with youth in primary care settings with large Māori populations. Objectives of the study are to evaluate the implementation of YouthCHAT in nurse-led youth clinics, school-based clinics, and general practice in Te Tai Tokerau (Northland, NZ); to develop a framework for the scaling up of YouthCHAT across further settings; to assess health provider and youth acceptability of the tool; to improve screening rates for mental health and help-seeking behavior; to enable early identification of emerging problems; and to improve brief intervention delivery.

**Methods:**

Using a bicultural mixed-methods co-design approach, 3 phases over a 3-year period will provide an iterative evaluation of the utility, feasibility, and acceptability of YouthCHAT, aiming to create a framework for wider-scale rollout and implementation.

**Results:**

Recruitment for the first phase began in September 2018. YouthCHAT was implemented at the first site in October 2018 and is expected to be at a further two sites in late January to early February 2019. The study is due for completion at the end of 2021.

**Conclusions:**

YouthCHAT has potential as a user-friendly, time efficient, and culturally safe screening tool for early detection of MHB issues in NZ youth. The resource pack assists the clinician to provide appropriate interventions for emerging and developed youth mental health and lifestyle issues. Involving input from community providers, users, and stakeholders will ensure that modifiable elements of YouthCHAT are tailored to meet the health needs specific to each context and will have a positive influence on future mental, physical, and social outcomes for NZ youth.

**International Registered Report Identifier (IRRID):**

PRR1-10.2196/12108

## Introduction

### Background

Mental health issues have overtaken cardiovascular disease and cancer as the leading cause of health loss in New Zealand (NZ) [1,2]. The majority of people affected by mental health concerns (eg, depression and anxiety) also exhibit more than 1 risk behavior (eg, smoking, problem drinking, substance abuse, and physical inactivity) [2]. These mental health and risk behaviors (MHBs) are the major contributing factors to NZ’s health loss [2]. The Ministry of Health recognizes that to improve the health of NZ, reduction of these MHBs is needed [2].

Most mental health disorders begin in adolescence [3-5], and risk behaviors established at this time can continue into adulthood [6-9], significantly impacting on long-term mental, physical, and social well-being [10-12]. Mental health issues affect 1 in 4 of NZ youth [4,13], and the number of NZ secondary school students reporting mood disorders, self-harm, and peer problems has increased [14]. NZ has the highest rate of youth suicide in the Organisation for Economic Cooperation and Development countries [15]. Māori (the indigenous people of NZ) youth living in deprived, rural areas have significantly higher rates of MHBs and suicide than their non-Māori peers. Further, educational, employment, and health outcomes are worse for adolescent Māori, and their life expectancy is much lower [16-21].

Rather than disclose their MHB concerns at primary care, adolescents often present primarily with vague, physical symptoms [8,9,16,22-25]. Opportunistic discussions about MHB do not occur in primary health care unless the problems are severe [9,26-34]. Yet, if the conversation is initiated by the clinician, young people are willing to discuss MHB [28,34-41]. Screening for youth MHB concerns occurs in less than 50% of primary health care provider (PHP) consultations, with the result that over half of adolescent MHB concerns are not detected [34,35,42]. Clinicians in primary care describe being underresourced in terms of time, appropriate screening tools, and experience in youth health [28,34,36]. Screening can reveal issues that may otherwise be overlooked and facilitate discussions between PHPs and young people [31]. This process can be therapeutic for youth, increasing their satisfaction with care and the likelihood that they will ask for help in the future [28,31,34].

There are a number of MHB screening tools available [41], but it can be time-consuming to administer these and interpret the results [43], and not all screening tools are suitable for use in all clinical contexts [43,44]. Where MHB screening for youth does occur, rates of follow-up and service use remain low [42,44,45]. The referral of every positive screen to secondary services creates an overwhelming need for appointments, delaying timely access to care [17,28,41,42,46]. In a stepped care approach, the primary health care team forms the hub of care for mild-to-moderate mental health issues. Increasing levels of care are added stepwise relative to the severity of more complex or worsening issues, providing effective management in primary care and more efficient use of specialist services [47].

The NZ-developed electronic Case-finding and Help Assessment Tool is a multi-item, electronic screening tool for use in primary health care to screen adults for substance misuse (smoking, alcohol, and drugs), problem gambling, depression, anxiety, exposure to abuse, difficulty controlling anger, and physical inactivity [48]. A youth version, YouthCHAT, has been developed using a co-design approach with input from clinicians and young people[49], and it contains 4 additional youth-specific modules: sexual health, youth stress, conduct disorder, and eating issues [10,48,50-55]. For positive screens in the smoking, substance abuse, depression, and anxiety modules, branching logic links users to validated assessment tools (eg, the Substances and Choices Scale [56], the Patient Health Questionnaire-Depression Adolescent version [57], and general anxiety disorder assessment, GAD-7 [58]). Some modules provide a nonclinical score that indicates the number of positive responses to questions (eg, the behavior or conduct module), and others simply indicate the presence of risky behavior (eg, gambling). The help question allows young people to prioritize concerns and indicate the areas where they are ready to accept help [51,59,60]. A stepped care-based resource pack guides clinicians to effective evidence-based interventions. Youth health providers in Northland have also undertaken mental health credentialing through the Primary health organisation (PHO) in order to provide brief interventions to clients for low to moderate mental health issues at the time of the screening.

Youth can complete YouthCHAT on an electronic device (eg, a tablet) before their consultation; completion takes approximately 5-15 minutes. A video briefly introduces the screen and explains confidentiality before giving the option to “start” or “exit” the screen. Upon completion of the screen, a clinical summary report is available to the clinician seeing them, indicating which domains screened positive, the severity of the condition (from validated assessments), and where help is sought. This report facilitates a conversation between the clinician and the young person, supporting shared decision making and youth input into their plan of care.

YouthCHAT has been successfully used in 1 rural NZ youth clinic and found to be acceptable to clinic staff (CS) and young people [54]. A randomized trial in an urban high school indicates that YouthCHAT compares favorably to the HEEADSSS (Home, Education, Eating, Activities, Drugs and Alcohol, Sexuality, Suicide and Depression, Safety) interview-based assessment, currently used to screen all Year 9 students in NZ low-decile secondary schools for psychosocial issues [49].

### Aim

This study will evaluate the implementation of the YouthCHAT program into primary health clinics in Te Tai Tokerau (Northland, NZ). Using a co-design approach, YouthCHAT, the processes around its use, and the stepped care resources offered will be tailored to meet the needs of local communities, with the aim of improving screening rates and brief intervention delivery for MHB concerns as well as developing a framework for scaling up the implementation of YouthCHAT more widely across Northland.

### Objectives

Specific objectives are to assess YouthCHAT with respect to the following:

1. Utility (does YouthCHAT lead to increased screening and detection rates of mental health issues?)
2. Feasibility (is it feasible to conduct e-screening and deliver stepped care support to young people attending youth clinics, school-based clinics, and general practice?)
3. Acceptability (is YouthCHAT acceptable to clinicians to use as a screening and stepped care intervention tool for youth?)
4. Provide a modifiable framework for scaling up implementation to meet specific health needs in specific contexts in consultation with community stakeholders, utilizing local agencies and cultural and community groups to deliver stepped care support.

## Methods

### Co-design and New Zealand Research

Considered the founding document of NZ, the Treaty of Waitangi was signed by Māori Chiefs and representatives of the British Crown in 1840. The agreement ratified the unification of Pākehā (Western settlers) and Māori as a nation while affording Māori values, traditions, and practices [61]. Inherent in the Treaty of Waitangi is the Māori principles of kāwanatanga (giving governance to the Crown) and tino rangatiratanga (respecting Māori self-determination). The Treaty of Waitangi Act in 1975 [62] outlined the principles of partnership, participation, and protection as representing the true intent of the Treaty [63-65]. The principles continue to be the foundation of the relationship between Māori and the New Zealand Government today [66]. Research involving Māori in NZ must be carried out in accordance with the principles of the Treaty of Waitangi and ensure that Māori culture, values, and beliefs are respected and protected [67-71].

A randomized controlled trial, considered the epitome of clinical trials, would provide evidence about how an intervention is used in a controlled context with a specific population. However, these findings may not apply to the use of the intervention with real populations in actual clinical settings [71-73], particularly in areas of wide cultural diversity, such as NZ [71].

Kaupapa Māori (research for the benefit of Māori by Māori) is influenced by Māori culture, history, indigenous knowledge (Mātauranga), language (te reo), values, and worldview [67,68,70,71,74-76]. The proverb “kaua e takahia te mana o te tangata” (do not trample on “mana” or dignity and status) highlights the guiding principle of Kaupapa Māori, to respect the mana not only of individuals but also of the Māori culture, values, and beliefs [67,68,77]. The self-determination of Māori to identify challenges, solve problems, and form new knowledge (tino rangatiratanga) affords control over research processes and the protection of Mātauranga [67-71]. This study was not conceived as a Kaupapa Māori project; however, as the majority of participants are envisaged to be Māori, their culture, values, and beliefs must be validated, respected, and protected. Provided Māori retain control of the research, non-Māori researchers can undertake research involving Māori. To do so, they must first reflect on their own culture, worldview, values, and beliefs and, having done so, acknowledge, give credence to, and respect those of Māori [67-71,77]. Non-Māori researchers can then work in partnership with Māori, as long the methodology affiliates with the principles and values of Kaupapa Māori and is undertaken in collaboration with those who will benefit from the research [78].

In a co-design approach, research is undertaken with, rather than on*,* people [67,79]. The research processes evolve in conjunction with and from the perspectives of the participants [79-82], allowing researchers to gain an understanding of context-specific requirements and challenges [79-84]. Participants become actively involved in the research process as partners, designers, and developers alongside the research team. The knowledge, experiences, ideas, and skills of the participants are used to help find acceptable and workable ways of identifying and overcoming problems [80,85,86]. Co-design research, therefore, aligns with Kaupapa Māori, and in this study, researchers from both cultures will work alongside each other sharing research principles, processes, and skills in bicultural research [87].

Māori have been involved in the development of YouthCHAT. The tool has been translated into te reo Māori (Māori language) and tested to ensure acceptability among Māori youth and clinicians in Te Tai Tokerau [54]. The same clinical educator (CE) who was part of the proof of concept trial noted above is a qualified nurse educator and registered nurse. As a coinvestigator and the Kaitautoko (support person or advocate) for this project, she will implement YouthCHAT in her clinical practice first and then train and work with other clinical staff as YouthCHAT is iteratively implemented in other clinics. Further, this research will be developed through engagement with young people, local iwi (confederation of Māori tribes), the local PHO and District Health Board, CS, and community providers for their input and participation. The cultural relevance of YouthCHAT and its implementation has been valued in its development. Equitable approaches will be taken with all participants in all locations, and the findings of this study may contribute to a long-term reduction in disparities between Māori and non-Māori young people. Engaging and partnering with stakeholders and users throughout the research process will respect Māori tikanga (customs and etiquette), and validation of Māori culture, values, and beliefs will be integral to the success of the study [75,82]. With the assistance of local project investigators, the bicultural, co-design approach will ensure that the YouthCHAT model initially employed, further scaled up, and then rolled out is culturally appropriate and meets the needs of the local community.

### Procedures

This study has received approval from the New Zealand Health and Disability Ethics Committee (reference 18/CEN/31) on May 05, 2018. A pragmatic rollout of YouthCHAT will take place over 3 years using an iterative process of implementation, modification, and evaluation to assess the processes for using YouthCHAT across different clinical settings in Te Tai Tokerau.

#### Whiringa Tuatahi Tahi (Phase 1)

After consultation with local health providers and other community resources, YouthCHAT will be implemented in 5 nurse-led youth clinics. Utility, feasibility, and acceptability data will be gathered and used to modify and improve YouthCHAT operational processes. The implementation of the YouthCHAT tool will be evaluated at each site. In response to the feedback received from participants at each clinic, modifications will be made to YouthCHAT processes.

#### Whiringa Tuatahi Rua (Phase 2)

After the changes from phase 1 have been incorporated and after further consultation with local providers and resources, using the updated implementation processes, YouthCHAT will be rolled out to 4 school-based clinics in Northland. Utility, feasibility, and acceptability data will be gathered and modifications made as before.

#### Whiringa Tuatahi Toru (Phase 3)

After the changes from phase 2 have been incorporated and after further consultation with local providers and resources, the updated implementation processes will then be used to roll out YouthCHAT to 3 general practice clinics in Northland. Utility, acceptability, and feasibility data will be gathered as before. The data from all 3 phases will be used to develop an implementation framework for a wider rollout of the YouthCHAT program across different primary health care settings in NZ.

### Setting, Participants, and Recruitment

The YouthCHAT program is being implemented into the youth screening policies and procedures across all the clinics taking part in this study. Youth between 12-24 years of age visiting the clinic will be asked to complete YouthCHAT as per the clinic’s screening procedures. Feedback about YouthCHAT will be sought in the form of surveys, interviews, and focus groups from 3 participant groups: (1) the CE, (2) CS, and (3) young people aged 16-24 years who have used YouthCHAT.

All CS, including the CE, will be informed about the study and invited to participate by members of the research team before the implementation of YouthCHAT into their practice. All youth meeting the participation criteria will be informed about and invited to take part in the study after their consultation with the clinician. This will be done by either the clinician or by administration staff, depending on each clinic’s systems. Young people who are interested in taking part will leave their contact details and will be contacted by the research team to arrange a suitable time for the focus groups. All participation is voluntary. Written, informed consent will be obtained prior to their participation. Youth taking part in the focus groups will receive a NZ $20 voucher, and kai (food) will be provided.

#### Inclusion Criteria

The study participants will include youth (over 16 years old) who have used YouthCHAT, CS from participating clinics who use the YouthCHAT tool, and the YouthCHAT CE, who provides training and support in the use of YouthCHAT.

#### Exclusion Criteria

Youth under 16 years old will be excluded as parental consent would have to be sought, and since young people often visit clinics without parental or guardian knowledge, seeking such consent would violate their confidentiality. Youth over 16 who are not able to provide their own consent (eg, due to cognitive difficulties) will be excluded.

### Data Collection

#### Utility Data and Measures

Data pertaining to each participating clinic’s screening rates for mental health issues and risky health behaviors and early identification of emerging problems and delivery of brief interventions before and after YouthCHAT implementation will be gathered by way of a summary report from the clinic administrator. The collated deidentified data will enable the calculation of screening rates and help-seeking behavior before and after the implementation of YouthCHAT. For all YouthCHAT modules, a simple “yes” or “no” metric will be used to determine rates of detection. Rates of health-seeking behavior will be assessed via the “help” question, which offers “yes,” “no,” and “yes but not today” responses that will be collected.

#### Acceptability and Feasibility Data

Acceptability and feasibility data will be gathered via surveys and interviews with the CE, CS, and focus groups with a subset of youth who have used YouthCHAT. Survey and interview questions will be framed around the mechanisms of normalization process theory (NPT): (1) coherence, for example, does it make sense for participants to implement YouthCHAT into their practice? (2) cognitive participation, for example, are participants able to easily engage with the tool? (3) collective action, for example, how hard is it to implement and use YouthCHAT? and (4) reflexive monitoring, for example, are there clear benefits of using YouthCHAT? Are the advantages or disadvantages of using the tool easily identifiable and how could disadvantages be minimized? [88-90].

Surveys and semistructured interviews with CS will gather feedback on their experience of using YouthCHAT, for example, perceptions of its usefulness, whether they would recommend it to others, and concerns about privacy and identify aspects that limit or encourage the use of YouthCHAT in their daily practice. A CE semistructured interview will be conducted at the end of data collection to discuss program feasibility, utility, acceptability, and suggested improvements in the setup processes of YouthCHAT and in training for its use.

Focus groups will be held with youth participants to gather in-depth feedback on their experience including the acceptability of the electronic method, perceptions of the look of the electronic interface, and the length of screening; whether they would recommend it to others; and concerns about privacy. Interviews and focus groups will be audiotaped and transcribed with consent.

### Data Handling

All information will be kept confidential, and analyzed data will be presented in a deidentified manner. HealthLink will provide secure storage of all YouthCHAT screening data and transmit encrypted, secure data to the clinics’ practice management system software by the same private network used for their electronic referrals. Participant data will be deidentified before being stored by the research team. Clinical data obtained through YouthCHAT will be available to relevant health providers. All surveys are Web-based and anonymous and will be completed using Qualtrics. Recordings of semistructured interviews and focus groups will be transcribed (without anything that could identify participants), and the recordings will be deleted. The transcriber will be asked to sign a confidentiality agreement. Data will be retained for 10 years as per the University of Auckland protocol.

### Data Analyses

#### Quantitative Data

Quantitative data will be analyzed using Microsoft Excel and a statistical software package. Analyses will include basic descriptive statistics (eg, number of youth screened and YouthCHAT summary data). Cross-tabulation analyses will explore differences in detection rates between different population subgroups (eg, ethnicity, age, and gender) across sites and compare pre-and post-YouthCHAT implementation. Subgroup analyses of differences in help-seeking behavior, uptake of the initiative, and delivery of interventions will also be analyzed if there are sufficient numbers to permit such analysis. This information will provide insights into important heterogeneities in our population of interest that may aid an effective intervention strategy.

#### Qualitative Data

Focus groups and semistructured interviews will be audiotaped and confidentially transcribed, and suggestions will be categorized and tabulated. Any modification to the implementation processes will follow majority opinion. Data will be analyzed using a general inductive approach [91], with collated text categorized and thematically analyzed according to the mechanisms of NPT. Data will be independently coded by 2 researchers, with a consensus reached by adjudication [88-90].

Presentation of the results will depend on the nature of the data collected. If substantial qualitative data allows, a thematic analysis will be conducted and presented separately. Irrespective of this, the results will be synthesized in the discussion.

## Results

Recruitment for the first phase began in September 2018. YouthCHAT was implemented at the first site in October 2018 and is expected to be at a further two sites in late January to early February 2019. The study is due for completion at the end of 2021.

## Discussion

### Expected Findings

Early detection intervention for mental health disorders and risky behaviors in youth can prevent long-term health and psychosocial issues [9,30,41,92]. For this to happen, it is imperative that a means of detecting existing and developing MHBs in adolescence is found and timely management initiated [22]. Interventions need to respect the ability of youth to change risk behaviors and provide them with the resources, skills, and support to do so [6,17,18,22,31,36,60,93,94]. YouthCHAT promotes wellness planning and supports youth to self-manage. The systematic approach of screening and provision of guides for stepped care intervention aims to lead to the early and more comprehensive intervention of youth mental health, substance misuse, and other lifestyle issues, which will have substantial impacts on future physical, mental, and social health as well as well-being. It is anticipated that a successful rollout of YouthCHAT will be associated with improved health and social outcomes through early identification of and intervention for mental health concerns, leading to an improvement in youth help-seeking behavior.

Complex interventions, such as YouthCHAT, with the potential to improve health outcomes, can only do so if they can be successfully implemented and then integrated or normalized into routine clinical practice [88-91]. However, this will not happen unless those who will be using the tool find it acceptable and unless its delivery is feasible. The implementation of YouthCHAT requires the necessary internet capability and availability of mobile devices, such as tablets, for the completion of the screen. The feasibility of implementing YouthCHAT relies on more than technological infrastructure, it is dependent on the impact YouthCHAT has on the people who will be using it [88-90]. For example, if clinical staff see the benefit of using YouthCHAT in their daily practice, enhancing engagement with young people and improving health outcomes while reducing their workload, then it is feasible that the tool can become normalized in their daily practice [88,89]. Feasibility is also reliant on factors that encourage or inhibit the uptake of the screen by young people, for example, the length of the screen, ease of completion, and the electronic interface. A co-design approach will assist the research team to identify and overcome barriers to the use of YouthCHAT.

With the assistance of local project investigators, the co-design, bicultural approach will ensure that the YouthCHAT model initially employed, further scaled up, and then rolled out, is culturally appropriate, meets the needs of the local community, and is easily integrated into practice. This study, underpinned by NPT, seeks to develop YouthCHAT as a sustainable, culturally acceptable, cost-effective, and time-saving screening tool for clinicians in primary care to identify mental health concerns and ultimately improve equity for Māori youth. This is particularly important in rural NZ where provider recruitment and retention remain difficult.

### Conclusions

If YouthCHAT can be implemented across a variety of primary health care settings in NZ, it has potential to be a user-friendly, time efficient, and culturally safe screening tool for early detection of psychosocial issues in NZ youth. The resource pack assists clinicians to provide appropriate intervention for emerging and developed youth mental health and lifestyle issues. The bicultural approach involving input from community providers, users, and stakeholders will ensure that modifiable elements of YouthCHAT can be tailored to meet the specific health needs of each context and could have a positive influence on future mental, physical, and social outcomes for NZ youth.

## Abbreviations

CE: clinical educator
CS: clinical staff
MHB: mental health and risk behavior
NPT: normalization process theory
NZ: New Zealand
PHO: primary health organization
PHP: primary health care provider

## Appendix

Multimedia Appendix 1 Health Research Council of New Zealand peer review report.
