# Supplementary material for: YouthCHAT as a Primary Care E-Screening Tool for Mental Health Issues Among Te Tai Tokerau Youth: Protocol for a Co-Design Study
Source: JMIR Res Protoc. 2019 Jan 9;8(1):e12108. doi: 10.2196/12108 (PMC6329425; doi:10.2196/12108)
Supplement: Multimedia Appendix 1 [file resprot_v8i1e12108_app1.pdf]

hr Health Research Council of New Zealand

26 October 2017

Professor Felicity Goodyear-Smith
Department of General Practice and Primary Health Care
School of Population Health
Faculty of Medical and Health Sciences
The University of Auckland
Private Bag 92019
Victoria Street West
Auckland 1142

Dear Felicity

2017 Global Alliance for Chronic Diseases - Application for Health Research Council Funding
HRC Reference: ***17/705***
Primary care e-screening for mental health among TeTai Tokerau youth

The Health Research Council of New Zealand (HRC), with the Global Alliance for Chronic Diseases (GACD), has completed the assessment of all proposals for the 2017 GACD RFP focussed on implementation research of mental health. Your proposal received independent assessment by three expert members of the joint assessment panel prior to the meeting. As a resulf of a good rank it was discussed at the full assessment meeting.

I am very pleased to advise that your proposal has been successful. The Council has offered funding to the level set out on the attached draft Third Schedule Summary.

Please note that budgetary changes to your original application may necessitate a change in your research objectives. These changes, or other enquiries relating to the administrative aspects of your funding, should be directed to your Research Office. Once all changes have been agreed contract documents will be sent to your Research Office for signing. A contract will not be formed until the HRC receives a completed_"Staff Declaration - HRC Contracts Form" (available from your research office) and copies of amended objectives and timelines and, any special conditions or requirements set out in the draft Third Schedule have been met. The *HRC Rules*, which form part of the contract, is available on the HRC website.

Some key conditions of the contract include best efforts to complete the proposed research, fulfilment of reporting requirements noting problems or delays as soon as they occur, changes or significant absences of key staff and significant changes to research objectives/ milestones. Regular reporting aims to identify any issues or concerns as well as highlight positive outcomes of the research. Please let us know directly of any newsworthy impacts of our funding. Contract variations, such as time extensions, must be submitted to the HRC by your research office. All research reports can be now submitted on the HRC Gateway.

Your host institution has been requested to accept contract offers by 31 November 2017. Unless your Research Office has received written authority from the HRC, your contract must commence no later than 28 February 2018. The funding may be withdrawn and returned to the HRC funding pool if this condition is not met.

Please note that the HRC will be making a media announcement about the outcome of this round mid-November 2017. Media activities initiated by your institution may follow the HRC's announcement but must not be before this announcement. This includes posting any result details on your websites. Please contact the HRC if you would like us to provide comment for your institution's media funding announcements as we would be happy to do so.

Some of the points raised during the assessment of your application are enclosed. If you wish to discuss the result of your application please address your enquiry, in the first instance, to your host institution and request that they write to Dr Deming Gong, Manager Research Investment -Contracts, at the HRC.

Note that all investigators receiving contract funding from the HRC must make themselves available, as reviewers or assessing committee members whenever possible. Please update your HRC Gateway profile to nominate yourself for HRC assessing committee membership.

I would like to add a personal note of congratulations on your success and I look forward to hearing of the progress and outcomes of your research.

Yours sincerely

Professor Kathryn McPherson
Chief Executive

enc Review report
Draft Third Schedule
Draft Fourth Schedule

c.c. Auckland UniServices, Research Office

APPLICATION: GACD-#532 HRC 17/705 Goodyear-Smith, "Primary care e-screening for mental health among Te Tai Tokerau youth"
OUTCOME: Discussed at assessment meeting, scored Very Good overall and recommended for funding within available budget.

Introducer #1

Relevance & quality

- the project aligns with the HRC call
- the scientific review is quite thorough, though it was expected that there should be some reference to similar work among indigenous populations in Australia or Canada
- the proposed methods are consistent with an implementation research approach, including iterative evaluation and revision of the tool
- the intervention focuses on the tool itself--more could be said about recognizing its limits
- ethics has been considered.

Quality of team

- this inter-disciplinary team includes an economist, health information specialist and a statistician. There is no evidence that the members have worked together before, so no track record of team performance is available
- only one "learner" is mentioned:-a "mature" PhD student
- a key "user" is involved
- Maori stakeholders are engaged in focus groups and several team members are Maori as well.

Feasibility

- not much is said about challenges and potential pitfalls
- some contextual factors are mentioned--such as high prison rates of Maori youth, and economic issues
- inequities regarding Maori youth are described in the literature review
- evaluation processes are included in the iterative approach; more specific evaluation plans could be shown to assess feasibility, acceptability and utility of the tool.

Potential impact

- the literature review lists several national commitments to this issue, such as the Mental Health Commission Blueprint II, and the NZ Suicide Prevention Action Plan: 2013-2016
- leverage with Maori councils is implied in two letters of support
- plans for step-wise scaling up is described for the duration of the project, but not sure after that.

Other comments

- the budget looks reasonable and is adequately justified
- Is there an on-going national network or professional organization focused on indigenous (Maori, etc.) health research issues, where relevant work can be shared among peers?

Introducer #2

Relevance and quality of project

The proposal addresses an important topic of Maori youth mental health, with a focus on detecting risk taking behaviour and mental health concerns among youth in primary care settings using a youth version of an electronic Case-Finding and Help Assessment Tool.

The approach used in the proposal could be strengthened by more fully considering barriers to care from a systems perspective, including acceptability not only of the tool itself, but also the cultural appropriateness of the existing primary care services for Maori youth, their

families, and communities.

While it is positive that the proposal references responsiveness to Maori, and mentions Health Research Council criteria for "Maori centred research," it would be helpful for the proposal to be more explicit about how these principles may have informed this project. The tool was previously evaluated with Maori youth, translated into the Maori language, and tested to ensure acceptability among Maori youth in the clinic setting. While the methods are described as co-design participatory implementation science research approach, and there is reference to engaging local iwi, there does not appear to be a clear governance structure to ensure that Maori community leadership have meaningfully informed decision-making in developing the proposal, and would do so throughout its implementation.

From the information provided, it is not clear whether Maori-specific ethical issues or governance aspects have been addressed from a community perspective.

The project focuses on help-seeking and behaviour change of individuals in primary care, using validated screening tools originating from non-Maori contexts. It is not clear whether Maori community input informed the tools used to date. The proposal would have been strengthened by consideration ofroot causes of youth challenges, and more fulsome consideration of strength-based approaches, including sense of identity and belonging. Maori "issues" and "viewpoints" are mentioned briefly, but it would have been helpful to have more holistic consideration of how the project could be strengthened by consideration of Maori worldview and culturally-specific determinants of health, including in the context of colonization and its impacts.

Quality of team

It is positive that there is reference to increasing Maori research capacity, and that the team includes a Maori researcher (Dr Clark). The information includes that Dr Clark completed a three-year training scholarship on social determinants of health in Indigenous populations; this broader perspective could more fulsomely inform the proposal. The track record of this team for engagement with Maori, and specifically regarding mental health, is not clear from the information provided.

One of the researchers led the development of the tool, which did not originate in a Maori context. The proposal does not mention the potential for power imbalances between Western and non-Western worldviews and the need for these to be addressed. Maori youth had been engaged in translating the tool. Although the tool is described as acceptable to youth, it is not clear if youth had opportunity to date to provide meaningful input into tool content. Roll-out is described as "pragmatic" and does not appear to be informed by stakeholder input. Although there is reference to the importance oflocal community input to success of interventions in general, and plans include engaging local iwi, methods include that the data are ultimately to be analysed by Maori researcher rather than youth and community leadership. The proposal would benefit from inclusion of a clear governance structure to further balance Western and Maori perspectives and strengthen implementation.

Feasibility of project

In response to the challenge of differences between local contexts, it is positive that modifiable elements of the intervention include adding local cultural and community supports into the stepped care intervention package; and community input in response to socioeconomic and contextual factors of specific regions. In terms of evaluation and data-gathering, the proposal would be strengthened by including information not only focused on individual risks and referrals to services, but also considering social determinants of health; more fulsome inclusion of data on individual strengths and cultural identity; and families, communities, and broader systems considerations such as organizational change. It would be helpful to know if data gathered would include client and/or family perspectives on the intervention or services received. The proposal would be strengthened by inclusion of Maori community input on Maori-specific, perhaps more holistic, aspects of evaluation and how

data might be collected and shared in ways that are accountable to and could support the broader community. Beyond encouraging help-seeking, it is not clear how the intervention helps promote youth identity and strengths. Gender could also be more clearly taken into account.

Potential impact
In terms of alignment with commitments, the proposal briefly mentions several policy documents that point to more integrated services for youth, with inter-sector collaboration; and the need to reduce inequity among Maori youth, which appear consistent with the overall approach. The extent to which local Maori community feedback informs the intervention would determine the extent to which scaled-up implementation would fit within local supports and services, and in relation to cultural, economic, and policy contexts. The project appears to have good potential impact. Scale up and potential benefit to the community could be significantly strengthened by increased involvement of Maori community and/or cultural leadership and confirmation of their formal endorsement and inclusion in the governance and decision-making of the project. The proposal mentions economic assessment, and includes a health economist on the team.

Introducer #3
Relevance and quality of project
The proposed research aligns strategically with the remit set by the funder. The proposed research will build on a solid platform of current evidence to inform an implementation plan that includes a co-design participatory approach with the target youth community and key stakeholders.

Quality of team
This is a formidable team with demonstrated track record in their respective fields, and experience in effective ongoing engagement in community settings with large Maori populations (Auckland and Northland). Importantly, the research team come with the strong support of the Northland kura kaupapa schools and local service provider which augurs very well for the regard held by the local community for the quality of the team and community willingness to work with the research team. The quality of this team is reflected in their research proposal.

Feasibility
The strength of the research team adds value to the feasibility of the proposed project. It builds on a solid platform for implementation research that identifies the need for scientific research and clearly sets out a realistic and therefore a pragmatic and manageable plan to achieve its overall aim and objectives. The proposed time commitment of the research team satisfactorily meets their respective roles and responsibilities.

Potential impact
The application directly aligns with both international and national commitments, appropriately leveraging on current knowledge with clear focus on users of the knowledge generated by the proposed research.

Panel Discussion Summary
This is an application from a strong team working with the community. The proposal could have more clearly detailed the context for implementation. There were questions regarding governance, recruitment of participants, effectiveness of the tool (although trialled in the

general community), and what was the linkage from screening to care. The panel was divided with respect to the quality of the research.

THIRD SCHEDULE SUMMARY - RESEARCH ACTIVITY DETAILS AND FUNDING

DRAFT

**Host:**	Auckland UniServices
**Contract Type:**	.Project GACD
**Contract Number:**	17/705
**First Named Investigator**	Professor Felicity Goodyear-Smith

Named Investigators: Mrs Rhiannon Martel, Dr Terryann Clark, Dr Margot Darragh, Professor Gail Pacheco, Dr Aniva Lawrence

Title: Primary care e-screening for mental health among TeTai Tokerau youth

Proposed Start Date: 1/07/2017 **Completion Date:** **Term:**

Actual Start Date:

Organisations Sharing in Funding:

Reporting Dates: Annually on the anniversary of the grant plus 1 month

Budget Note:

Date Printed: 24-0ct-2017

**Budget Outline (GST Exclusive)**	$
**Total Budget:**	624,349.00
**Administered by HRC:**	0.00
**Host Budget:**	624,349.00

**Key Personnel**	
Professor Felicity Goodyear-Smith	0.10
Dr Terryann Clark	0.05
Professor Gail Pacheco	0.03
Dr Aniva Lawrence	0.10
Mrs Rhiannon Martel	1.00
Dr Margot Darragh	0.20
**Total FTE:**	**1.48**

Personnel marked * have a time commitment only

Monthly Payment:

Payment Process:

monthly on the 2oth day of the month

FOURTH SCHEDULE -
RESEARCH OBJECTIVES AND MILESTONES

DRAFT

Note that this page will form the basis of the contract for progress reports

#	Ob"ectives
1	Engaging with local stakeholders to identify possible improvements to YouthCHAT through staged rollout, using iterative rocess of im lementation & evaluation
2	Assessing feasibility & acceptability of YouthCHAT in nurse-led youth clinics, school-based clinics & general practice in Te Tai Tokerau
3	Identifying utility of YouthCHAT in nurse-led youth clinics, school-based clinics & general practice in Te Tai Tokerau
4	Identifying changes in screening rates for risky health behaviours, mental health, help-seeking behaviour, early identification of emer in roblems & intervention deliver
5	Developing framework for scaling up implementation of YouthCHAT, including cost-benefit analysis of wider rollout

Ye r/#'.for.CorhbletiorfofMilestonesfoteac:ti Pbiective		
Year#t.,	Milestones	t,•,,pbjective'{s)P.\
1	Ethical Approval	1,2,3,4
1	Phase 1 rollout commenced	1
1	Phase 1 data collection complete	2
1	YouthCHAT 2.0 launched	1
1	Phase 2 rollout commenced	2
2	Phase 2 data collection complete	2,3,4
2	YouthCHAT 3.0 (if applicable) developed	1
3	Phase 3 data collection complete	2
3	Data integrated and analysed	3,4
3	Development of framework	

Date Printed: 24-0ct-2017
